# Genome-Wide Analysis of Sheep Artificially or Naturally Infected with Gastrointestinal Nematodes

**DOI:** 10.3390/genes14071342

**Published:** 2023-06-26

**Authors:** Jacob W. Thorne, Reid Redden, Scott A. Bowdridge, Gabrielle M. Becker, Morgan R. Stegemiller, Brenda M. Murdoch

**Affiliations:** 1 Department of Animal, Veterinary and Food Sciences, University of Idaho, Moscow, ID 83844, USA; jake.thorne@ag.tamu.edu (J.W.T.);; 2 Texas A&M AgriLife Research and Extension, San Angelo, TX 76901, USA; 3 Division of Animal and Nutritional Sciences, West Virginia University, Morgantown, WV 26506, USA

**Keywords:** sheep, gastrointestinal nematodes, fecal egg count, genome-wide association

## Abstract

The anthelmintic resistance of gastrointestinal nematodes (GINs) poses a significant threat to sheep worldwide, but genomic selection can serve as an alternative to the use of chemical treatment as a solution for parasitic infection. The objective of this study is to conduct genome-wide association studies (GWASs) to identify single nucleotide polymorphisms (SNPs) in Rambouillet (RA) and Dorper × White Dorper (DWD) lambs associated with the biological response to a GIN infection. All lambs were genotyped with a medium-density genomic panel with 40,598 markers used for analysis. Separate GWASs were conducted using fecal egg counts (FECs) from lambs (<1 year of age) that acquired their artificial infections via an oral inoculation of 10,000 *Haemonchus contortus* larvae (*n* = 145) or naturally while grazing on pasture (*n* = 184). A GWAS was also performed for packed cell volume (PCV) in artificially GIN-challenged lambs. A total of 26 SNPs exceeded significance and 21 SNPs were in or within 20 kb of genes such as *SCUBE1*, *GALNT6*, *IGF1R*, *CAPZB* and *PTK2B*. The ontology analysis of candidate genes signifies the importance of immune cell development, mucin production and cellular signaling for coagulation and wound healing following epithelial damage in the abomasal gastric pits via *H. contortus* during GIN infection in lambs. These results add to a growing body of the literature that promotes the use of genomic selection for increased sheep resistance to GINs.

## 1. Introduction

Gastrointestinal nematodes (GINs) are a critical threat to global sheep production, particularly from the highly pathogenic *H. contortus*. In the United States (USA), sheep are produced in a variety of systems and climates [1]. However, the overuse of the limited number of commercially available anthelmintics, even in more temperate and arid regions, has contributed to the resistance of GINs to dewormers nationwide [2,3,4]. Similar findings were reported in global parasite populations for nearly two decades [5]. The employment of rapidly developing genomic technology can be a key resource for elucidating the biological mechanisms behind the response to GINs and improving the natural resistance of sheep.

The Dorper, White Dorper and Rambouillet breeds are common in the USA, but multiple reports indicate that they are more susceptible to GINs than breeds of Caribbean descent [6,7,8,9]. A further understanding of the physiological mechanisms responsible for the host resistance to parasites in these breeds is needed for future directional selection to occur. With *H. contortus* regarded as the GIN of greatest concern, artificial parasite challenges were conducted in Dorper, Rambouillet and other breeds of sheep as a means to measure and improve the understanding of the biological response of lambs to this singular parasite [7,10]. Under natural grazing conditions, sheep are exposed to multiple GIN species that can be difficult to differentiate using standard fecal egg counting practices. Data from either a species-specific artificial challenge or less-controlled natural challenge can serve as a resource for genome-wide association studies (GWASs) to identify genomic markers of importance for parasite resistance.

Employing GWASs to identify single nucleotide polymorphisms (SNPs) for GIN response phenotypes has been a popular recent approach [11,12,13,14]. Genomic analyses with Dorper sheep specifically were reported [15,16,17], but Rambouillet were understudied using these same approaches. Not to mention, with the polygenic nature of parasite resistance and considering the within-breed diversity that likely exists, further work in these popular USA breeds is warranted. The objectives of this study are to perform GWASs to identify the SNP markers associated with parasite response phenotypes collected from lambs under either an artificial or natural GIN challenge.

## 2. Materials and Methods

### 2.1. Animal Background

Dorper × White Dorper (DWD) and Rambouillet (RA) lambs were all sourced from Texas A&M AgriLife Research flocks in San Angelo, TX, USA. From 2019 to 2022, during the post-weaning timeframe (sheep from the ages of 4 to 12 months), lambs from these two flocks were subjected to either an artificial *H. contortus* challenge (*n* = 145) or a natural parasite challenge (*n* = 184) while grazing on pasture. No lambs were included in both the artificial or natural challenge analyses.

All lambs were managed with their dams on pasture until they reached 75–90 days of age, at which point they were weaned and dewormed using Cydectin (0.2 mg/kg; Bayer Animal Health, Shawnee Mission, KS, USA) and Valbazen (7.5 mg/kg; Zoetis Inc., Kalamazoo, MI, USA), and then managed according to either the artificial or natural challenge protocols. All procedures were approved by the Texas A&M Agriculture Animal Care and Use Committee with Animal Use Protocol #2020-19A.

### 2.2. Artificial Parasite Challenge Data

In 2020, Rambouillet lambs (*n* = 81) were placed in two adjoining feedlot pens (30 m × 30 m), where they were provided grain ad libitum. The dirt feedlot pens did not contain grass, and thus, were considered a ‘GIN-free’ environment. After a 60-day adjustment period, all lambs were orally inoculated with 10,000 L3 *H. contortus*, and FECs and packed-cell volume (PCV) were recorded at 21 days post inoculation (dpi) and 35 dpi. Fecal samples were collected directly from the rectum and stored at 4 °C until analysis. All FECs were determined via a modified McMaster technique [18] that utilized a 2 g fecal sample homogenized in 28 mL of sodium nitrate solution (specific gravity = 1.25 M). Following mixing and removal of solids by straining through double-layered gauze, the solution was placed on a McMaster slide and strongyle eggs were counted under 100× magnification to a sensitivity of 50 epg. To determine PCV, whole blood was collected via the jugular venipuncture in 16 × 100 mm purple-top tubes containing EDTA and also stored at 4 °C. All samples were centrifuged at 4000 rpm for seven minutes, and hematocrit percentage was subsequently measured. For a comprehensive description of this study and the results, see [10].

In 2022, following the protocol previously employed with the RA lambs, DWD lambs (*n* = 64) were also subjected to an artificial *H. contortus* challenge in a feedlot setting. In contrast to the RA lambs, which were born in the spring and were reared by their dams on pastures during a season conducive to GIN survival, the DWD lambs were born in the late fall, and reared with their dams on pasture during the winter, which is a time of hypobiosis for *H. contortus*. Fecal samples from RA lambs at weaning indicated they had previous exposure (a primary challenge) to GINs. Given that not all DWD lambs may have had a primary exposure to *H. contortus* as was expected with RA lambs, all DWD lambs were inoculated with a dose of 2000 *H. contortus* L3 larvae and then were orally drenched with Prohibit (8 mg/kg; Agri Laboratories Ltd., St. Joseph, MO, USA) 10 d. later. After a two-week recovery period, lambs were orally inoculated with 10,000 *H. contortus* L3 larvae to initiate the artificial challenge trial. In line with sampling timepoints from RA lambs, FECs and PCV were recorded at 21 d and 35 d post infection for DWD lambs.

### 2.3. Natural Parasite Challenge Data

**Figure 1:**
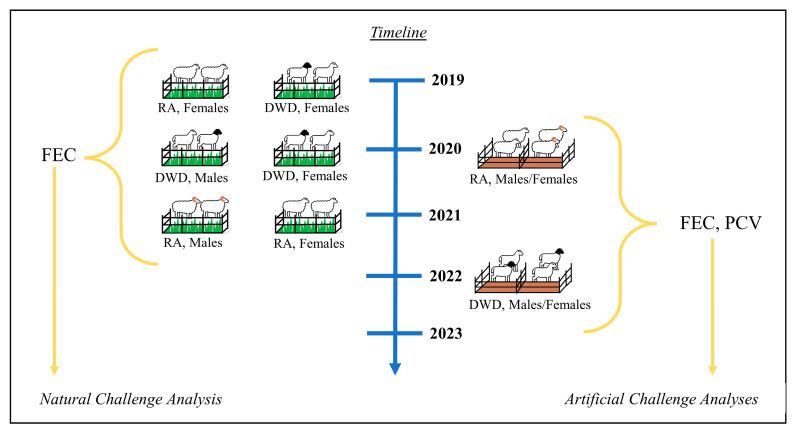
Visual portrayal of the design utilized in this project. Fecal egg counts (FECs) from six different contemporary groups of Rambouillet (RA) and Dorper × White Dorper (DWD) lambs naturally challenged with gastrointestinal nematodes were compiled for genome-wide association analyses. Fecal egg counts and packed-cell volume (PCV) were compiled from two separate artificial GIN challenges, either with RA lambs or DWD lambs.

Post-weaning FECs from multiple contemporary groups of RA (*n* = 90) and DWD (*n* = 94) lambs from 2019 to 2021 were compiled for analysis (Figure 1). All groups were managed under the same protocol, at weaning lambs were orally drenched with Cydectin and Valbazen and placed on pasture previously grazed by GIN-infected sheep. To allow for substantial time for lambs to become reinfected with GINs, fecal collections were not conducted for at least 60 d following deworming. Lambs were likely exposed to multiple GIN species on pasture, as was observed in similar studies [8], and the amount of parasites consumed by each individual could not be determined. In 2019, a coproculture analysis was performed for GIN speciation in RA grazing at the Texas AgriLife Research Station, and the results revealed 89% *H. contortus*, 10% Trichostrongylus and 1% Strongyloides. Further coproculture analyses were not performed in the following years.

The level of GIN contamination on pastures was not determined, but fecal sampling occurred during the warmer season (summer/fall, temperatures above 27 °C) when environmental conditions were favorable for *H. contortus*. Entire contemporary groups were also only individually fecal sampled once a subset of samples confirmed that the mean FEC of the group exceeded 500 eggs per gram (epg). Previous research indicated that a 500 epg FEC average indicates the occurrence of a moderate parasite challenge [19,20,21,22]. All FECs were performed using the McMaster method described previously.

### 2.4. Genotyping

All artificially and naturally infected lambs were genotyped with either the Axiom™ Ovine Genotyping 50 K Array (Thermo Fisher Scientific, Waltham, MA, USA) or the AgResearch Sheep Genomics 60 K SNP chip (GenomNZ, AgResearch, Mosgiel, New Zealand). Using SNP and Variation Suite (Golden Helix, Bozeman, MT, USA), a combined working dataset of 41,431 SNPs was generated by retaining genomic markers that overlapped between the two panels, with the remaining SNPs being discarded. Using PLINK v1.90, quality control filtering was performed for call rate (>90%), minor allele frequency (>99%), Hardy–Weinberg equilibrium (1.0 × 10^−6^) and the removal of duplicates, resulting in 40,598 SNPs remaining for analysis.

### 2.5. Statistical Analyses

To meet normality, FEC data from both the artificial and natural challenges were BoxCox transformed (TFEC) in R v 4.0.3, and GWASs were performed using PLINK v1.90 [23,24]. In both the artificial and natural analyses, phenotypic data from the two different breeds of sheep were combined to increase the power of the GWAS. Recognizing the need to account for across- and within-breed population structures, a principal component (PC) analysis was conducted via PLINK, and the top 20 PCs were fitted in the model as covariates. To ensure proper population stratification, the genomic inflation factor (λ) was calculated via PLINK, and all GWAS models reported had λ = 1.

In the artificial challenge analysis, additive and non-additive models were tested against FEC and PCV at 21 dpi, 35 dpi and the rate of change between these two timepoints. Reported are phenotypes for which significant results were obtained, which included GWAS with FEC and PCV at 35 dpi (recessive model) and a rate of change for both FEC (additive model) and PCV (recessive model) between 21 dpi and 35 dpi. The rate of change was determined by first scaling the phenotypes to a range of all positive values to accommodate the regression analysis, and then using the slope of the line fit between the two timepoints.

For the natural challenge analyses, contemporary groups each consisted of lambs of one breed type and one sex (Figure 1). Individual sex, breed and year effects in the model could not be differentiated from one another, but to account for variable parasite levels that challenged the contemporary groups in different pasture environments, the ‘--family’ flag was used first to cluster the samples by group (which had six unique breed, sex and year combinations). Using a recessive model, GWAS was performed for TFEC with body weight and PC included as covariates. Manhattan plots displaying the GWAS results were developed using the ‘qqman’ [25] and ‘dplyr’ [26] packages in R. Significance was determined through permutation testing of GWAS models (50,000 replications) using PLINK and set at 2.0 × 10^−5^ for the artificial challenge analyses and 6.0 × 10^−5^ for the natural challenge analysis.

Linkage disequilibrium between pairs of significant SNPs identified was calculated using the ‘--ld’ flag in PLINK v1.90, which computes a haplotype-based r2 statistic. Haplotype block identification was performed using the ‘--blocks’ flag, also in PLINK, with the default setting of identifying SNPs in strong LD (defined by [27]) within 200 kB of one another.

### 2.6. Gene Identification and Annotation

Reported SNP locations are from the ARS-UI_Ramb_v2.0 genome assembly [28]. Proximity of an SNP to a gene was explored using Genome Data Viewer from the National Center for Biotechnology Information (https://www.ncbi.nlm.nih.gov/genome/gdv/, accessed on 1 February 2023). Further analysis of candidate genes occurred if a significant SNP was located within 20 kB of the gene. Gene functional annotation and corresponding ontology (GO) terms for Ovis aries were sourced from the UniProt database [29]. In the instance that annotation in sheep was not available, gene function was subsequently sourced from the Bos taurus database. A heatmap visually depicting enriched biological processes was developed using ‘ggplot’ from the ‘tidyverse’ package in R [30].

## 3. Results

### 3.1. Artificial Challenge GWAS Results

**Figure 2:**
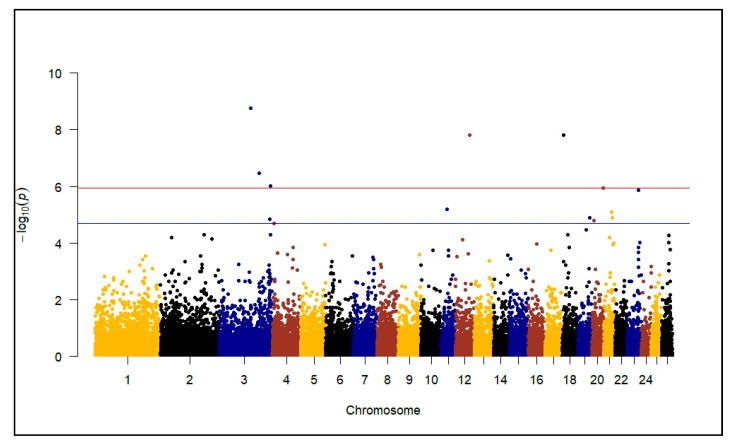
Manhattan plot displaying results of genome-wide association study (recessive model) with transformed fecal egg count (TFEC) 35 days post inoculation in an artificial *H. contortus* challenge trial. The blue line represents significance set via permutation testing at −log10 (2.0 × 10^−5^), but the plots also include a red line depicting the more stringent Bonferroni level of significance of −log10 (1.16 × 10^−6^).

Genome-wide significant SNPs associated with TFEC during a *H. contortus* infection were identified (Figure 2). The descriptive statistics of the FEC and PCV phenotypes by breed for which the associations were tested in artificially challenged lambs are displayed in Table 1, and the significant SNP marker information is described in Table 2. The significant SNPs were identified in intronic regions of *GALNT6*, *SYNGR1*, *CEP350*, *IGF1R*, *RHOA*, *ZBTB44*, *AHNAK* and *CTIF*.

**Figure 3:**
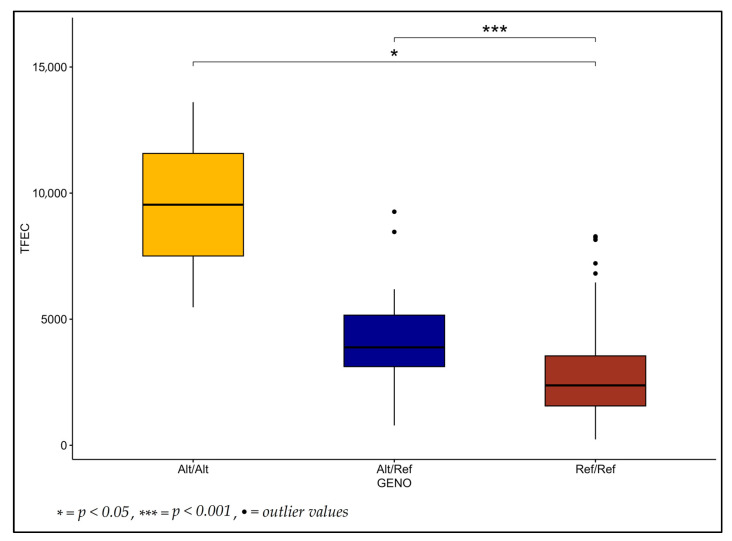
Transformed fecal egg count (TFEC) of lambs by genotype for rs159935395; an SNP located in exon 18 of *SCUBE1* identified using a recessive model in GWAS. Lambs homozygous for the reference allele had a reduced TFEC compared to heterozygous (*p =* 0.0005) and homozygous alternative allele lambs (*p =* 0.044). There were 2, 27 and 118 lambs in the ‘Alt/Alt’, ‘Alt/Ref’ and ‘Ref/Ref’ groups, respectively.

An SNP on chromosome 3 in exon 18 of *SCUBE1* was associated with increased TFEC (Figure 3). This variant allele at rs159935395 was predominantly present in the RA lambs (freq = 0.179) versus the DWD lambs (freq = 0.008), where it was only reported in one heterozygous Ref/Alt lamb. Furthermore, four additional SNPs were identified within 9 kb of the genes *DERL2*, *TULP1*, *PXDC1* and *LOC114114021*.

**Figure 4:**
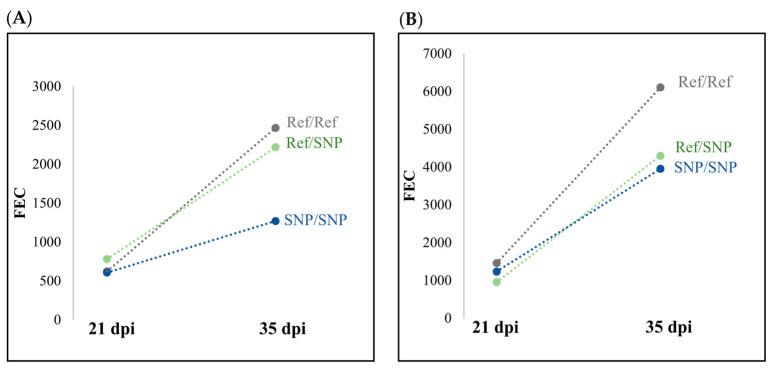
The FEC change between an early (21 d post inoculation) and established (35 d post inoculation) gastrointestinal nematode infection, plotted by genotype for the SNP rs415241061, which had the highest level of reported significance identified using GWAS conducted in a multibreed dataset. This SNP is in an intronic region of *CAPZB*, on chromosome 2, as described by the most current Ovis aries genome assembly, ARS-UI_Ramb_v2. (**A**) FEC change in Dorper × White Dorper lambs by genotype. (**B**) FEC change of Rambouillet lambs by genotype.

In contrast to the natural parasite challenge, the artificial parasite challenge protocol included repeated data collections over multiple timepoints, allowing for the analysis of phenotype change over the period of time when the GIN infection was expected to develop. When performing the GWAS for rate of change of the FECs from 21 dpi of the artificial challenge to 35 dpi, concisely described as FEC slope, two more significant SNPs were identified when an additive model was employed. One of these markers, rs415241061, is located in an intron region of *CAPZB*. In both breeds of sheep, the lambs homozygous for the alternate SNP had a reduced FEC slope (Figure 4).

In addition to the FEC phenotypes, the PCV was also captured during the artificial challenge trials as a quantification of the lamb resilience to *H. contortus* infection. No SNPs meet significance for the PCV at 21 dpi with the GWAS; however, at 35 dpi, three significant SNPs were identified, including two intronic SNPs in *SLC49A4* and *GLCE*. When analyzing the PCV slope, two significant SNPs were identified on chromosome 2, with rs422296454 being located in exon 6 of *TRIM14*.

### 3.2. Natural Challenge GWAS Results

The descriptive statistics of the phenotypic information for each of the six groups included in the natural challenge dataset are described in Table 3. The mean FEC for each group ranged from 835 epg to 1919 epg, indicating that the lambs were exposed to moderate parasite challenges. Using a recessive model, six significant SNPs were identified. As described in Table 4, two of these SNPs were associated with a decrease in TFEC, and three were associated with an increase in TFEC. Three of the identified SNPs, on chromosome 12, were located in intronic regions of the genes *EXO1, BRINP3* and *DNM3*.

The frequencies of significant SNPs within each breed type are provided in Table 4, with frequencies ranging from 0.022 to 0.489 for the RA lambs, and 0.229 to 0.484 for the DWD lambs. Two SNPs on chromosome 12 associated with an increase in TFEC, rs429291496 and rs417624219, in DWD lambs, have the same frequency. Linkage disequilibrium (LD) analysis, which shows that they have an r^2^ value of 1, indicates that they are in full LD (Supplementary Table S1). In the RA lambs, these two SNPs had an r^2^ value of 0.49. The follow-up haplotype analysis revealed that these two SNPs are included in a five-SNP haplotype block spanning 147 kb in the DWD lambs (Supplementary Figure S1). The genes located within this block include *MAP1LC3C*, *EXO1* and *WDR64*.

### 3.3. Gene Ontology

**Figure 5:**
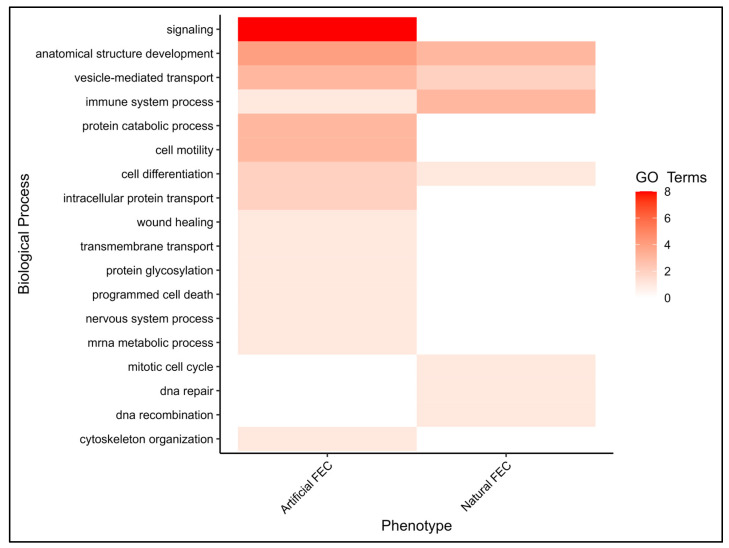
Heatmap depicting biological processes enriched by gene ontology terms (GO Terms) associated with positionally significant candidate genes from either the artificial or natural parasite challenge analysis. Candidate genes were within 20 kb of significant SNPs identified through GWASs with FECs.

When searched in the UniProt database, the genes identified via the GWASs returned 110 unique GO terms (Supplementary Table S2). The associated biological processes (BPs) for the GO terms associated with positionally significant genes identified for each phenotype are reported in Figure 5. Across all the phenotypes, ‘signaling’ and ‘anatomical structural development’ were the BPs with the greatest number of subprocesses associated with the candidate genes revealed in this study.

## 4. Discussion

With the data captured from two separate experimental procedures, this study utilizes GWASs to identify the markers associated with the biological response of lambs from two sheep breeds to GIN infection. Both the DWD and RA lambs in this study are from breeds that are common for lamb production in the USA, but were previously described as more susceptible to GINs than other breeds with more known resistance [7,31,32,33]. While we do not assume that the response of the RA and DWD lambs to GINs are exactly the same, the data from the two breed types were combined for both the artificial and natural challenge analyses to increase the sample population. When each breed was analyzed individually, few significant results were returned; however, this could have been due to a limited sample size for each breed. Furthermore, due to the restricted scope of this study, it is important to consider the limited number of breeding rams used (eight RA sires and seven DWD sires) in this project. The SNP and gene variant frequencies that exist in the RA and Dorper or White Dorper breeds may not be comprehensive in this study.

In total, the GWASs identified 20 significant SNPs associated with the FECs and PCV collected from the lambs under an artificial *H. contortus* challenge, and 6 significant SNPs associated with lamb FECs when collected following a natural parasite challenge. Of the 26 significant SNPs identified in this study, 21 were located in exons, introns or within 20 kb of genes, suggesting that *SCUBE1, TRIM14, EXO1*, *BRINP3*, *DNM3*, *GALNT6*, *CEP350*, *IGF1R*, *SYNGR1*, *RHOA*, *ZBTB44*, *AHNAK*, *CTIF*, *CAPZB*, *PTK2B*, *DERL2*, *TULP1*, *PXDC1, SLC49A4*, *GLCE* and *LOC114114021* all potentially influence the lamb response to GINs in the populations we evaluated.

Multiple markers within the genes were identified in the GWASs for the FECs of lambs artificially challenged with *H. contortus*, including one SNP in the exon of *SCUBE1*. Signal peptide CUB domain and EGF-like domain containing 1, encoded by *SCUBE1*, is a member of the epithelial growth factor superfamily and is highly expressed in platelets [34] and vascular endothelial cells [35]. More specifically, it is a cell surface glycoprotein that is thought to assist in platelet aggregation, as observed in mice [36]. In our artificial parasite challenge, lambs received a large dose of *H. contortus* larvae in a single inoculation, which all likely reached maturity and began feeding on blood simultaneously. Previous abomasal transcriptome research revealed the increased expression of genes involved in the complement and coagulation pathways in merino lambs artificially infected with *H. contortus* larvae [37].

The SNP with the highest significance identified in our study when tested with FEC at 35 dpi, rs424235017 (*p* = 1.80 × 10^−9^), is located within the intron of *GALNT6*. Al Kalaldeh et al., 2019 [38] also identified SNPs within *GALNT6* associated with FECs in a large GWAS that included Dorper and Merino sheep, in addition to other breeds. This result is also in line with that of Benavides et al., 2015 [16] whose GWAS with FECs in Dorper and Red Maasai sheep identified an SNP marker within ~100 kb of a gene within the same family as *GALNT4*. A KEGG analysis indicates that the *GALNT* family of genes are paramount in the Mucin type O-glycan biosynthesis pathway, which is important for modifying the serine or threonine residues of proteins. Mucins are highly glycosylated proteins and are a primary component of the mucosa layer that serves as an initial barrier against helminths attempting to burrow into the gastric pits of the abomasum [39]. GIN-susceptible sheep parasitized with *H. contortus* larvae were shown to have reduced and altered types of mucins present in the abomasal mucosa layer, but not in more GIN-resistant animals [40]. The mutation of *GALNT6* observed in our study may be associated with a decrease in the mucin production or glycosylation, resulting in a greater establishment of *H. contortus*.

Another SNP with high significance in our study was located in *IGF1R*, which encodes the insulin-like growth factor 1 receptor. IGF1R binds IGF with a high affinity and is critical for cell growth and survival as it is an upstream activator of the PI3K-AKT/PKB and Ras-MAPK pathways. Previous research has identified associations between variants of *IGF1R* and increased growth in sheep [41,42,43]. Berton et al., 2017 [44] identified an SNP in *IGF1R* as being associated with hematocrit in naturally parasitized Santa Inês sheep. Chen et al., 2012 [45] identified increased levels of *Igf-1*expression in mice artificially infected with helminths. In addition, Chen et al., 2012 [45] found increased levels of IL-4 and IL-13, which are hallmark cytokines for a Th2-type immune response and promoters of localized wound healing [46].

Multiple quantitative trait loci (QTL) associated with FEC in Merino sheep were identified upstream of our identified SNP from 107.3 to 119.9 Mbp on chromosome 2 [38]. In addition, another intronic SNP on chromosome 2, rs415241061, exceeded the significance threshold in our study when testing for FEC changes. This marker is located within *CAPZB*, which encodes an F-actin capping protein that is important in muscle development. Hong et al., 2017 [47] also revealed that in mice, CAPZB binds to gp96, which is a member of the heat shock protein 90 chaperones, providing a potential link between CAPZB and innate immune function.

The remaining genes with an intronic SNP identified in the GWASs with artificial challenge FEC phenotypes include *CEP350*, *SYNGR1*, *RHOA*, *ZBTB44*, *AHNAK* and *CTIF*. To our knowledge, these genes have not been previously reported to be linked to parasite resistance in sheep. CEP350 plays a role in stabilizing microtubules in the Golgi apparatus of animal cells [48]. SYNGR1 is critical in the presynaptic vesicle formation in neurons and is notably associated with brain disorders in humans [49]. *RHOA* is a member of the Rho family that plays a role in cellular signal transduction. Mutations in *RHOA* were also associated with T follicular helper cell specification [50], and one of the leading GO terms associated with *RHOA* is GO:0044319, which is also associated with wound healing and the spreading of cells. *ZBTB44* is a member of the zinc finger and BTB domain-containing family, whose functions include wide ranging B- and T-cell development [51]. *AHNAK* encodes a large nuclear phosphoprotein that also impacts TGFβ signaling [52], and which can ultimately have downstream immune function effects. *CTIF* is critical for the pioneer round of mRNA translation and gene expression [53].

In the artificial challenge GWAS analyses, two significant SNPs in the intron regions of *SLC49A4* and *GLCE* were associated with a PCV at 35 dpi, almost exclusively in the RA lambs. Disrupted in renal cancer protein 2 (*DIRC2*) is an alias of *SLC49A4* and encodes a metabolite transporter; previous associations to renal tumor formation were described in humans with mutant *DIRC2* [54]. *GLCE* encodes the glucuronic acid epimerase enzyme, which plays an active role in the glycosaminoglycan biosynthesis–heparan sulfate/heparin metabolic pathway, which was shown to be enriched in a previous GWAS exploring parasite resistance in Morada Nova sheep [14]. During the synthesis of heparan sulfate proteoglycans (HSPGs), GLCE converts glucuronic acid to iduronic acid [55], which, in turn, promotes the binding ability of HSPGs [56]. While HSPGs have a multitude of biological functions, it was previously reported that HSPG mutant mice have reduced mast cell and platelet aggregation [57].

In addition, two significant SNPs associated with PCV change exceeded significance in the GWAS, including a marker in exon 6 of *TRIM14*, which encodes the tripartite motif 14 protein. TRIM14 is believed to have a wide range of biological roles, including affecting the innate immune response to viral infection [58]. An additional SNP in an intronic region of *PTK2B* was also identified in our study. *PTK2B*, also known as PYK2B, is a tyrosine kinase that is commonly expressed in hematopoietic cells and plays an essential role in platelet aggregation [59]. A mutation in *PTK2B* could limit the ability of lambs in this study with abomasal epithelial hemorrhage to coagulate at the wound-site, though further research would be needed to confirm this theory.

Three genes, *EXO1*, *BRINP3* and *DNM3*, identified with the natural challenge data, were all located on chromosome 12, which harbors multiple previously identified QTLs associated with FECs in several breeds of sheep [60,61,62]. Exonuclease 1 (EXO1) was described as important for genome maintenance, playing a central role in Mre11-Rad50-Xrs2 recruitment and cellular regulation during DNA double-stranded break repair [63,64]. Mice with double-knockout *Exo1* were shown to have a significantly higher cancer predisposition and 50% lower survival rate at 16 months [65]. In our results, we observed an incremental increase in the TFEC per allele of the rs429291496 SNP in *EXO1*, suggesting that this SNP may be associated with gene function. *EXO1* is more highly expressed in mesenteric lymph nodes, lymph node prescapular and Peyer’s patch compared to other tissues in sheep, insinuating that it has a role in immune function [66]. A previous study identified a QTL for FECs in French breeds of sheep that is located within 1 Mb of rs429291496, when mapped to the OAR v3.1 assembly [61].

Curiously, in DWD lambs only, rs429291496 was in full LD with four other SNPs covering a 147 kb span from position 34,187,202 to 34,334,816 of chromosome 12. Upstream of *EXO1* in this haplotype block includes *MAP1LC3C,* which plays an important role in autophagy and cellular maintenance [67]. Downstream of *EXO1*, but still within the same block, includes *WDR64*; however, it is almost exclusively expressed in the testes [65]. The complete haplotype block identified in the DWD lambs was not present in the RA lambs in this study.

Furthermore, *BRINP3* is predominantly a regulator of neuron differentiation, and knockout studies in mice have shown that *Brinp3-/-* mice have altered sociability [68]. Interestingly, the under-expression of *BRINP3* in humans was also associated with Ulcerative Colitis [69]. Also highly expressed in the central nervous system is *DNM3*, which is involved in microtubule formation and vesicular transport and was reported to be down regulated in human cases of colon cancer [70].

The functions of the positional candidate genes identified using either the artificial or natural parasite challenge GWASs were further explored in this study. The biological processes associated with the three fully annotated genes revealed in the natural challenge analyses included ‘anatomical structural development’, ‘immune system processes’, ‘vesicle mediated transport’ and ‘cell differentiation’. Despite no candidate genes identified in common between the natural and artificial GWAS, all four of these biological processes were also enriched by GO terms associated with genes revealed exclusively in the artificial analyses. While ‘anatomical structural development’ is a broadly defined term, this BP was also highly enriched in the analyses, suggesting that tissue regeneration is a critical component of withstanding a parasite infection.

Including both natural and artificial parasite infections in this study, as well as focusing on two parasite-susceptible breeds with distinct characteristics, provided a multiplicative approach to identifying SNPs, candidate genes and physiological differences that may differentiate the ability of sheep to withstand GINs. It is important to reiterate that in the natural parasite challenge, the lambs were potentially exposed to multiple GIN species, but likely at a lower and more consistent rate than the lambs in the artificial challenge, which were inoculated with a single large dose of *H. contortus* larvae at a given time point. The difference in the design of the natural and artificial trials (‘trickle’ infection with potentially mixed species vs. one-time dose of *H. contortus* only), in addition to the fact that the same lambs were not subjected to both protocols, may contribute to why the variation in the genomic regions and positional candidate genes did not overlap. Even with some incongruencies between the infection scenarios and the fact that the candidate genes revealed in the analyses differed, there is evidence that there is an overlap in the physiological response to GINs regardless of how the parasites are consumed (Figure 5). It is also evident from the annotation analyses that the ability of lambs to mobilize cellular resources to reconstruct tissue when withstanding a one-time inoculation with *H. contortus* is important.

Given the multiple gene regions identified in this study, as well as in other studies, it is unlikely that significant progress for improved resistance in sheep will be achieved by selecting for individuals with a single preferred gene variant or SNP genotype. Genetic selection for multiple preferred haplotypes with a larger effect on GIN resistance phenotypes or even genomically enhanced breeding values that fit the effects of numerous SNPs will be necessary for rapid progress. Additional consideration may be given to crossbreeding strategies, which could combine beneficial gene variants for parasite resistance, as this study identified novel candidate genes that were not previously discovered in other breeds with a similar research design. Further research with a larger sample size that is more robust against the founder effect may be able to more clearly delineate the individual breed differences that may exist between the RA and DWD lambs.

## 5. Conclusions

These analyses revealed 26 significant genomic markers for parasite susceptibility in hair and wool breeds of sheep when challenged either naturally or artificially with GINs. *H. contortus* remains a significant health challenge in Dorper, White Dorper and Rambouillet sheep, and our results support the consensus that the susceptibility to this GIN is polygenic and variable across and within breed type.

Importantly, for future research, significant SNPs identified in this study also provide insight into the physiological mechanisms that are responsible for the resistance or susceptibility to GINs. Our results further reiterate the importance of effective cellular signaling to aid not only in a timely immune response for the host defense against GINs, but in the efficient regeneration of tissue following parasite infection. Furthermore, we identified markers that can serve as a foundation and resource for future parasitology research within these breeds and be used in concert with other markers for directional selection towards animals that are better equipped to withstand parasite challenges.

## Figures and Tables

**Table 1:** Descriptive phenotype statistics of the Dorper × White Dorper (DWD) and Rambouillet (RA) lambs that were inoculated with *H. contortus* larvae during an artificial parasite challenge.

	*n*	FEC (epg) 35 dpi	FEC (epg) Change from 21 to 35 dpi	PCV (%) 35 dpi	PCV (%) Change from 21 to 35 dpi
DWD	64	1931 ± 176	626 ± 83	34.97 ± 0.45	−0.47 ± 0.14
RA	81	4440 ± 262	1657 ± 118	30.76 ± 0.45	−1.18 ± 0.15

**Table 2:** Genome-wide association of significant SNPs with *H. contortus* response phenotypes collected during an artificial parasite challenge trial.

Phenotype	Model	SNP rsID	Chr	Position	Unadj. *p*-Value	Ref/Alt	DWD Alt. Freq	RA Alt. Freq	Effect on Pheno.	Nearest Gene	SNP Proximity to Gene
FEC 35 dpi	Rec	rs424235017	3	134,890,798	1.80 × 10^−9^	G/A	0.125	0.223	Increase	*GALNT6*	Intron
		rs401156132	3	169,841,077	3.38 × 10^−7^	T/C	0.164	0.259	Increase	*LOC114114021*	6409 bp 3′
		rs404143758	3	216,603,922	1.43 × 10^−5^	C/T	0.086	0.410	Increase	*SYNGR1*	Intron
		rs159935395	3	219,954,194	9.88 × 10^−7^	C/T	0.008	0.181	Increase	*SCUBE1*	Exon (18 of 23)
		rs429438214	11	25,967,278	6.28× 10^−6^	G/A	0.070	0.217	Increase	*DERL2*	715 bp 5′
		rs405220677	12	60,593,402	1.54 × 10^−8^	A/G	0.000	0.229	Increase	*CEP350*	Intron
		rs413192275	18	7,290,863	1.54 × 10^−8^	G/T	0.047	0.325	Increase	*IGF1R*	Intron
		rs419813974	19	50,763,869	1.26 × 10^−5^	T/C	0.109	0.205	Increase	*RHOA*	Intron
		rs415404019	20	9,607,339	1.58 × 10^−5^	C/T	0.000	0.229	Increase	*TULP1*	6025 bp 3′
		rs410477651	20	49,324,023	1.15 × 10^−6^	T/C	0.086	0.217	Increase	*PXDC1*	8627 bp 5′
		rs424286945	21	33,794,983	8.10 × 10^−6^	C/T	0.031	0.229	Increase	*ZBTB44*	Intron
		rs415721024	21	37,387,654	1.27 × 10^−5^	C/A	0.109	0.265	Increase	*AHNAK*	Intron
		rs409725836	23	48,687,824	1.39 × 10^−6^	C/T	0.273	0.342	Increase	*CTIF*	Intron
FEC change	Add	rs415241061	2	246,106,891	6.45 × 10^−6^	G/A	0.352	0.605	Decrease	*CAPZB*	Intron
		rs401640382	26	23,345,876	1.60 × 10^−5^	T/C	0.367	0.114	Increase	*LONRF1*	47,248 bp 5′
PCV 35 dpi	Rec	rs419089993	1	188,044,970	9.28 × 10^−6^	A/G	0.008	0.187	Decrease	*SLC49A4*	Intron
		rs406909457	7	16,032,244	5.59 × 10^−6^	A/G	0.000	0.217	Decrease	*GLCE*	Intron
		rs400878817	15	58,581,288	5.59 × 10^−6^	T/C	0.000	0.151	Decrease	*LOC114118298*	68,801 bp 5′
PCV change	Rec	rs430428851	2	38,404,704	6.26 × 10^−6^	T/C	0.500	0.370	Increase	*PTK2B*	Intron
		rs422296454	2	49,663,835	5.86 × 10^−6^	G/A	0.102	0.235	Increase	*TRIM14*	Exon (6 of 6)

**Table 3:** Dorper × White Dorper (DWD) and Rambouillet (RA) lambs’ contemporary group descriptive statistics from which fecal egg counts were compiled for the natural parasite challenge GWAS. Each group of lambs was managed on a separate pasture.

Year	Breed	Sex	*n*	Mean FEC ± s.e.
2019	DWD	F	30	1730 ± 506
2020	DWD	F	31	1919 ± 87
2020	DWD	M	33	835 ± 85
2019	RA	F	16	1363 ± 137
2021	RA	F	47	840 ± 93
2021	RA	M	17	1117 ± 203
Total			184	1252 ± 106

**Table 4:** Genome-wide association with significant SNPs associated with fecal egg count in Dorper × White Dorper (DWD) and Rambouillet (RA) lambs.

SNP rsID	Chr	Position	Unadj. *p*-Value	Ref/Alt.	DWD Alt. Freq	RA Alt. Freq	SNP Effect	Nearest Gene	SNP Proximity to Gene
rs428558490	3	92,426,733	1.44 × 10^−5^	A/C	0.229	0.022	Increase	*LOC101102137*	29,816 bp 5′
rs425919895	11	4,259,584	2.00 × 10^−5^	A/G	0.405	0.011	Decrease	*LOC101118198*	294,502 bp 3′
rs415220805	12	14,701,365	2.00 × 10^−5^	G/A	0.484	0.356	Decrease	*BRINP3*	Intron
rs417624219	12	34,187,202	3.87 × 10^−5^	G/A	0.452	0.489	Increase	*MAP1LC3C*	23,836 bp 5′
rs429291496	12	34,257,171	4.23 × 10^−6^	G/A	0.452	0.356	Increase	*EXO1*	Intron
rs422997699	12	39,106,959	3.59 × 10^−5^	C/T	0.325	0.172	Decrease	*DNM3*	Intron

## Data Availability

The data presented in this study are available upon request from the corresponding authors. The data are not publicly available due to privacy restrictions of the genotyping array platforms.

## Supplementary Materials

The following supporting information can be downloaded at: https://www.mdpi.com/article/10.3390/genes14071342/s1, Figure S1: A 147 kb region of interest, containing 5 SNP with r^2^ = 1; Table S1: Linkage disequilibrium between SNP from natural parasite challenge GWAS; Table S2: Gene ontology biological processes.
