# Supplementary material for: Genome-Wide Analysis of Sheep Artificially or Naturally Infected with Gastrointestinal Nematodes
Source: Genes (Basel). 2023 Jun 26;14(7):1342. doi: 10.3390/genes14071342 (PMC10379027; doi:10.3390/genes14071342)
Supplement: Supplementary file 1 [file genes-14-01342-s001.zip › Supplemental Figure S1.pdf]

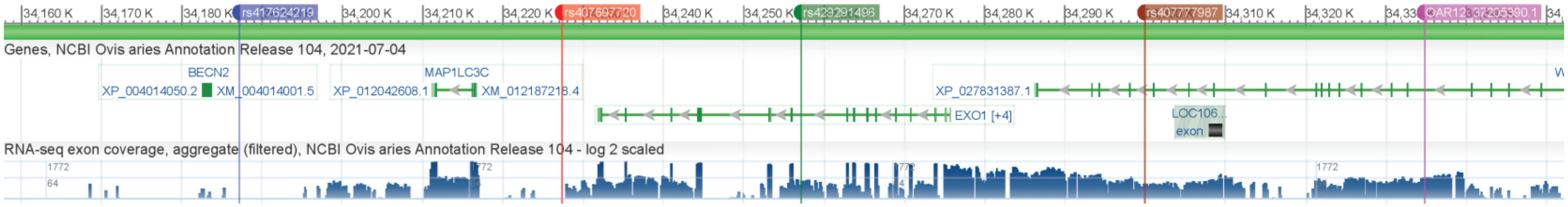


**Supplemental Figure S1**. A 147 kb region of interest, containing 5 SNP with $r^2=1$ was identified in Dorper x White Dorper lambs associated with an increase in FEC. Included within this region are the genes *MAP1LC3C*, *EXO1* and *WDR64*. Image was created using the Genome Data Viewer from the National Center for Biotechnology Information (https://www.ncbi.nlm.nih.gov/genome/gdv/browser/genome/?id=GCF_016772045.1 )
