# Supplementary material for: Genome-Wide Analysis of Sheep Artificially or Naturally Infected with Gastrointestinal Nematodes
Source: Genes (Basel). 2023 Jun 26;14(7):1342. doi: 10.3390/genes14071342 (PMC10379027; doi:10.3390/genes14071342)
Supplement: Supplementary file 1 [file genes-14-01342-s001.zip › Supplemental Table S1.pdf]

Supplemental Table S1. Linkage disequilibrium between significant SNP identified in GWAS with natural parasite challenge FEC. Values in the table were calculated with haplotype-based r^2 method in PLINK v1.90.

SNP1	SNP2	DWD	RA
rs415220805	rs417624219	0.035	0.012
rs415220805	rs429291496	0.035	0.011
rs415220805	rs422997699	0.094	0.020
rs417624219	rs429291496	1.000	0.490
rs417624219	rs422997699	0.001	0.107
rs429291496	rs422997699	0.001	0.028
