# Supplementary material for: Genome-Wide Analysis of Sheep Artificially or Naturally Infected with Gastrointestinal Nematodes
Source: Genes (Basel). 2023 Jun 26;14(7):1342. doi: 10.3390/genes14071342 (PMC10379027; doi:10.3390/genes14071342)
Supplement: Supplementary file 1 [file genes-14-01342-s001.zip › Supplemental Table S2.pdf]

Supplemental Table S2. List of gene ontology biological processes and corresponding term IDs associated with candidate genes identified in both the artificial and natural parasite challenge GWAS.

Analyses	Phentoype	Candidate Gene	Gene Ontology Biological Process Term	Gene Ontology ID
Art. Challenge	FEC 35 dpi	*AHNAK*	regulation of RNA splicing	GO:0043484
Art. Challenge	FEC 35 dpi	*AHNAK*	regulation of voltage-gated calcium channel activity	GO:1901385
Art. Challenge	FEC 35 dpi	*CEP350*	microtubule anchoring	GO:0034453
Art. Challenge	FEC 35 dpi	*CEP350*	protein localization to centrosome	GO:0071539
Art. Challenge	FEC 35 dpi	*CEP350*	non-motile cilium assembly	GO:1905515
Art. Challenge	FEC 35 dpi	*CTIF*	nuclear-transcribed mRNA catabolic process, nonsense-mediated decay	GO:0000184
Art. Challenge	FEC 35 dpi	*CTIF*	regulation of translational initiation	GO:0006446
Art. Challenge	FEC 35 dpi	*DERL2*	suckling behavior	GO:0001967
Art. Challenge	FEC 35 dpi	*DERL2*	positive regulation of cell population proliferation	GO:0008284
Art. Challenge	FEC 35 dpi	*DERL2*	positive regulation of cell growth	GO:0030307
Art. Challenge	FEC 35 dpi	*DERL2*	ubiquitin-dependent ERAD pathway	GO:0030433
Art. Challenge	FEC 35 dpi	*DERL2*	endoplasmic reticulum unfolded protein response	GO:0030968
Art. Challenge	FEC 35 dpi	*DERL2*	retrograde protein transport, ER to cytosol	GO:0030970
Art. Challenge	FEC 35 dpi	*DERL2*	negative regulation of retrograde protein transport, ER to cytosol	GO:1904153
Art. Challenge	FEC 35 dpi	*GALNT6*	protein O-linked glycosylation via threonine	GO:0018243
Art. Challenge	FEC 35 dpi	*IGF1R*	immune response	GO:0006955
Art. Challenge	FEC 35 dpi	*IGF1R*	positive regulation of cell population proliferation	GO:0008284
Art. Challenge	FEC 35 dpi	*IGF1R*	positive regulation of cell migration	GO:0030335
Art. Challenge	FEC 35 dpi	*IGF1R*	peptidyl-tyrosine autophosphorylation	GO:0038083

Art. Challenge	FEC 35 dpi	*IGF1R*	negative regulation of apoptotic process	GO:0043066
Art. Challenge	FEC 35 dpi	*IGF1R*	negative regulation of MAPK cascade	GO:0043409
Art. Challenge	FEC 35 dpi	*IGF1R*	transcytosis	GO:0045056
Art. Challenge	FEC 35 dpi	*IGF1R*	regulation of JNK cascade	GO:0046328
Art. Challenge	FEC 35 dpi	*IGF1R*	phosphatidylinositol-mediated signaling	GO:0048015
Art. Challenge	FEC 35 dpi	*IGF1R*	amyloid-beta clearance	GO:0097242
Art. Challenge	FEC 35 dpi	*IGF1R*	cellular response to amyloid-beta	GO:1904646
Art. Challenge	FEC 35 dpi	*RHOA*	small GTPase mediated signal transduction	GO:0007264
Art. Challenge	FEC 35 dpi	*RHOA*	wound healing, spreading of cells	GO:0044319
Art. Challenge	FEC 35 dpi	*RHOA*	skeletal muscle satellite cell migration	GO:1902766
Art. Challenge	FEC 35 dpi	*TULP1*	phagocytosis, recognition	GO:0006910
Art. Challenge	FEC 35 dpi	*TULP1*	dendrite development	GO:0016358
Art. Challenge	FEC 35 dpi	*TULP1*	eye photoreceptor cell development	GO:0042462
Art. Challenge	FEC 35 dpi	*TULP1*	photoreceptor cell maintenance	GO:0045494
Art. Challenge	FEC 35 dpi	*TULP1*	positive regulation of phagocytosis	GO:0050766
Art. Challenge	FEC 35 dpi	*TULP1*	detection of light stimulus involved in visual perception	GO:0050908
Art. Challenge	FEC 35 dpi	*TULP1*	retina development in camera-type eye	GO:0060041
Art. Challenge	FEC 35 dpi	*TULP1*	protein localization to photoreceptor outer segment	GO:1903546
Art. Challenge	FEC 35 dpi	*SCUBE1*	positive regulation of smoothened signaling pathway	GO:0045880
Art. Challenge	FEC Slope	*CAPZB*	actin polymerization or depolymerization	GO:0008154
Art. Challenge	FEC Slope	*CAPZB*	lamellipodium assembly	GO:0030032
Art. Challenge	FEC Slope	*CAPZB*	barbed-end actin filament capping	GO:0051016
Art. Challenge	PCV 35 dpi	*GLCE*	heparan sulfate proteoglycan biosynthetic process	GO:0015012

Art. Challenge	PCV 35 dpi	*GLCE*	heparin biosynthetic process	GO:0030210
Art. Challenge	PCV Slope	*PTK2B*	positive regulation of cell-matrix adhesion	GO:0001954
Art. Challenge	PCV Slope	*PTK2B*	sprouting angiogenesis	GO:0002040
Art. Challenge	PCV Slope	*PTK2B*	marginal zone B cell differentiation	GO:0002315
Art. Challenge	PCV Slope	*PTK2B*	signal complex assembly	GO:0007172
Art. Challenge	PCV Slope	*PTK2B*	integrin-mediated signaling pathway	GO:0007229
Art. Challenge	PCV Slope	*PTK2B*	positive regulation of cell population proliferation	GO:0008284
Art. Challenge	PCV Slope	*PTK2B*	negative regulation of cell population proliferation	GO:0008285
Art. Challenge	PCV Slope	*PTK2B*	regulation of cell shape	GO:0008360
Art. Challenge	PCV Slope	*PTK2B*	positive regulation of endothelial cell migration	GO:0010595
Art. Challenge	PCV Slope	*PTK2B*	regulation of cGMP-mediated signaling	GO:0010752
Art. Challenge	PCV Slope	*PTK2B*	regulation of macrophage chemotaxis	GO:0010758
Art. Challenge	PCV Slope	*PTK2B*	positive regulation of neuron projection development	GO:0010976
Art. Challenge	PCV Slope	*PTK2B*	negative regulation of bone mineralization	GO:0030502
Art. Challenge	PCV Slope	*PTK2B*	positive regulation of actin filament polymerization	GO:0030838
Art. Challenge	PCV Slope	*PTK2B*	regulation of inositol trisphosphate biosynthetic process	GO:0032960
Art. Challenge	PCV Slope	*PTK2B*	tumor necrosis factor-mediated signaling pathway	GO:0033209
Art. Challenge	PCV Slope	*PTK2B*	peptidyl-tyrosine autophosphorylation	GO:0038083
Art. Challenge	PCV Slope	*PTK2B*	activation of Janus kinase activity	GO:0042976
Art. Challenge	PCV Slope	*PTK2B*	negative regulation of apoptotic process	GO:0043066
Art. Challenge	PCV Slope	*PTK2B*	negative regulation of potassium ion transport	GO:0043267
Art. Challenge	PCV Slope	*PTK2B*	positive regulation of phosphatidylinositol 3-kinase activity	GO:0043552
Art. Challenge	PCV Slope	*PTK2B*	regulation of nitric oxide biosynthetic process	GO:0045428

Art. Challenge	PCV Slope	*PTK2B*	bone resorption	GO:0045453
Art. Challenge	PCV Slope	*PTK2B*	negative regulation of myeloid cell differentiation	GO:0045638
Art. Challenge	PCV Slope	*PTK2B*	positive regulation of angiogenesis	GO:0045766
Art. Challenge	PCV Slope	*PTK2B*	positive regulation of JNK cascade	GO:0046330
Art. Challenge	PCV Slope	*PTK2B*	vascular endothelial growth factor receptor signaling pathway	GO:0048010
Art. Challenge	PCV Slope	*PTK2B*	positive regulation of peptidyl-tyrosine phosphorylation	GO:0050731
Art. Challenge	PCV Slope	*PTK2B*	regulation of calcium-mediated signaling	GO:0050848
Art. Challenge	PCV Slope	*PTK2B*	positive regulation of nitric-oxide synthase activity	GO:0051000
Art. Challenge	PCV Slope	*PTK2B*	regulation of release of sequestered calcium ion into cytosol	GO:0051279
Art. Challenge	PCV Slope	*PTK2B*	chemokine-mediated signaling pathway	GO:0070098
Art. Challenge	PCV Slope	*PTK2B*	positive regulation of ERK1 and ERK2 cascade	GO:0070374
Art. Challenge	PCV Slope	*PTK2B*	cellular response to retinoic acid	GO:0071300
Art. Challenge	PCV Slope	*PTK2B*	cellular response to fluid shear stress	GO:0071498
Art. Challenge	PCV Slope	*PTK2B*	endothelin receptor signaling pathway	GO:0086100
Art. Challenge	PCV Slope	*PTK2B*	regulation of postsynaptic density assembly	GO:0099151
Art. Challenge	PCV Slope	*PTK2B*	positive regulation of ubiquitin-dependent protein catabolic process	GO:2000060
Art. Challenge	PCV Slope	*PTK2B*	regulation of establishment of cell polarity	GO:2000114
Art. Challenge	PCV Slope	*PTK2B*	regulation of actin cytoskeleton reorganization	GO:2000249
Art. Challenge	PCV Slope	*PTK2B*	positive regulation of B cell chemotaxis	GO:2000538
Art. Challenge	PCV Slope	*TRIM14*	innate immune response	GO:0045087
Nat. Challenge	PW FEC	*BRINP3*	positive regulation of neuron differentiation	GO:0045666
Nat. Challenge	PW FEC	*BRINP3*	negative regulation of mitotic cell cycle	GO:0045930
Nat. Challenge	PW FEC	*EXO1*	humoral immune response mediated by circulating immunoglobulin	GO:0002455

Nat. Challenge	PW FEC	*EXO1*	mismatch repair	GO:0006298
Nat. Challenge	PW FEC	*EXO1*	somatic hypermutation of immunoglobulin genes	GO:0016446
Nat. Challenge	PW FEC	*EXO1*	isotype switching	GO:0045190
Nat. Challenge	PW FEC	*DNM3*	receptor internalization*	GO:0031623
Nat. Challenge	PW FEC	*DNM3*	synaptic vesicle endocytosis*	GO:0048488
